# HCV Cure With Direct-Acting Antivirals Improves Liver and Immunological Markers in HIV/HCV-Coinfected Patients

**DOI:** 10.3389/fimmu.2021.723196

**Published:** 2021-08-23

**Authors:** Óscar Brochado-Kith, Isidoro Martínez, Juan Berenguer, Juan González-García, Sergio Salgüero, Daniel Sepúlveda-Crespo, Cristina Díez, Víctor Hontañón, Luis Ibañez-Samaniego, Leire Pérez-Latorre, Amanda Fernández-Rodríguez, María Ángeles Jiménez-Sousa, Salvador Resino

**Affiliations:** ^1^Unidad de Infección Viral e Inmunidad, Centro Nacional de Microbiología, Instituto de Salud Carlos III, Majadahonda, Madrid, Spain; ^2^Unidad de Enfermedades Infecciosas/VIH, Hospital General Universitario “Gregorio Marañón”, Madrid, Spain; ^3^Instituto de Investigación Sanitaria del Gregorio Marañón, Madrid, Spain; ^4^Unidad de VIH, Servicio de Medicina Interna, Hospital Universitario “La Paz”, Madrid, Spain; ^5^Instituto de Investigación Sanitaria La Paz (IdiPAZ), Madrid, Spain; ^6^Unidad de Análisis Clínicos, Hospital Universitario Fundación Alcorcón, Alcorcón, Spain; ^7^Servicio de Aparato Digestivo, Hospital General Universitario Gregorio Marañón, Madrid, Spain; ^8^Centro de Investigación Biomédica en Red de Enfermedades Hepáticas y Digestivas (CIBERehd), Madrid, Spain

**Keywords:** HIV/HCV coinfection, immune system, cirrhosis, DAA therapy, plasma biomarkers, PBMCs, gene expression

## Abstract

Hepatitis C virus (HCV) cure after all-oral direct-acting antiviral (DAA) therapy greatly improves the liver and immune system. We aimed to assess the impact of this HCV clearance on immune system-related markers in plasma and the gene expression profile in human immunodeficiency virus (HIV)/HCV-coinfected patients with advanced cirrhosis. We performed a prospective study on 33 HIV/HCV-coinfected patients at baseline and 36 weeks after the sustained virological response. Gene expression was evaluated by RNA-seq analysis on peripheral blood mononuclear cells (PBMCs) and plasma biomarkers by multiplex immunoassays. We found a decrease in plasma biomarkers (PD1, PDL1, CXCL10, CXCL8, IL12p70, IL10, and TGFβ) and liver disease markers (stiffness measurement (LSM), hepatic venous pressure gradient (HVPG), and transaminases, among others). Furthermore, decreased plasma levels of CXCL8, CXCL10, IL10, and PD1 were associated with reduced LSM values. We also found two upregulated (*HAS1* and *IRG1*) and 15 downregulated (*CXCL11, CCL8, CCL7, CCL2, ADARB2, RRAD, MX1, SIGLEC1, IFI44L, IFI44, IFI27, IFI6, IFIT3, IFIT1B*, and *IFIT1*) genes at the end of follow-up, all interferon-stimulated genes (ISGs) grouped into four pathways (“cytokine-cytokine receptor interaction”, “viral protein interaction with cytokine and cytokine receptor”, “chemokine signaling pathway”, and “hepatitis C”). Additionally, the decrease in most of these ISGs was significantly related to reduced LSM and HVPG values. In conclusion, HIV/HCV-coinfected patients with advanced-HCV-related cirrhosis who eradicated HCV following DAA therapy exhibited an improvement in liver disease markers and a significant decrease in plasma biomarkers and gene expression related to antiviral/inflammatory response, particularly in levels of several chemokines and ISGs.

## Introduction

Hepatitis C virus (HCV) infection causes chronic liver disease worldwide (1). Moreover, many people are coinfected with HCV and human immunodeficiency virus (HIV) (2). These individuals develop liver cirrhosis over decades that progress faster than in HCV-monoinfected patients (3). Likewise, HIV/HCV-coinfected individuals have higher rates of liver-related events (LRE) that included liver decompensation, chronic liver failure, hepatocellular carcinoma (HCC), liver transplantation, and liver-related deaths (4–7). Immunosuppression can explain this negative impact of HIV on chronic hepatitis C, but also because HIV can contribute to liver inflammation and fibrosis by the direct action of the virus itself on the hepatic stellate cells (8, 9), as well as factors related to lifestyle, such as alcohol consumption. Additionally, different extrahepatic manifestations associated with HCV, such as autoimmune, lymphoproliferative, metabolic, renal, cardiovascular, and central nervous system disorders, may also contribute to the morbimortality of HIV/HCV-coinfected patients (10).

HCV infection causes dysregulation of immune functions such as inflammation and immune activation and, together with other comorbidities, promotes cirrhosis progression and the development of LRE (7, 11, 12). Chronic HCV infection is characterized by an abundant hepatic and systemic production of inflammatory mediators and interferon-stimulated genes (ISGs) that cannot control the virus, indicating exhaustion of the interferon (IFN) system (13). Additionally, alterations in immune cells such as CD4^+^ and CD8^+^ T cells and natural killer (NK) cells follow HCV infection (14). For example, CD4^+^ and CD8^+^ lymphocytes show an exhausted phenotype with high levels of programmed death protein 1 (PD1), which stimulates their apoptosis (15–17). Also, a T-helper cell type 2 (Th2) predominance over a Th1 response has been proposed to promote chronic hepatitis C (18, 19). Moreover, HIV patients on suppressive antiretroviral therapy (ART) also show dysregulation of the immune system (7, 11), with very diverse alterations, including immune activation (20), inflammation (21), a deficit in T-cell functions (20, 22–26), and dysbiosis (27–29), which increase the risk of acquired immunodeficiency syndrome (AIDS), non-AIDS-related events, and death (30, 31).

Direct-acting antivirals (DAA) have transformed HCV therapy because almost all treated patients achieve a sustained virological response (SVR) (32–35). HCV eradication after DAA therapy decreases the risk of LRE in HCV-monoinfected patients with compensated cirrhosis, although this decrease has not been observed among those with decompensated cirrhosis (36–38). HIV/HCV-coinfected patients have a similar risk of LRE after successful DAA therapy as HCV-monoinfected individuals; however, the former group has a higher chance of non-liver-related death (39, 40). All this makes it necessary to study and monitor the cirrhotic patient after HCV eradication.

Peripheral blood transcriptome data provide crucial information on host immune response against pathogens, including HCV infection (41). For example, transcriptome analysis following successful DAA therapy offers a unique opportunity to analyze the possible normalization of the host response after years of chronic infection (42, 43). So far, only a few studies have explored the long-term impact of HCV clearance from DAA therapy on the peripheral blood transcriptome of HIV/HCV-coinfected patients (44, 45).

### Objective

We aimed to evaluate the impact of HCV eradication following all-oral DAA therapy on immune system-related markers in plasma and the gene expression profile in peripheral blood mononuclear cells (PBMCs) in HIV/HCV-coinfected patients with advanced cirrhosis.

## Patients and Methods

### Study Subjects

We carried out a prospective study on 33 HIV/HCV-coinfected patients with advanced HCV-related cirrhosis who started anti-HCV therapy with all-oral DAA from four hospitals in Madrid (Spain) between January 2015 and June 2016 (ESCORIAL study; see **Appendix**). Our study was approved by the Research Ethics Committee of the Instituto de Salud Carlos III (CEI PI 41_2014) and was conducted according to Helsinki’s Declaration. All participants gave their signed written consent at the start of the study.

The inclusion criteria were: 1) chronic HCV and HIV infection; 2) prior history of liver decompensation (bleeding esophageal varices, ascites, hepatic encephalopathy) or existence of advanced cirrhosis (hepatic venous pressure gradient (HVPG) ≥10 mmHg, liver stiffness measurement (LSM) ≥25 kPa, or Child-Turcotte-Pugh (CTP) ≥7); 3) starting all-oral DAA therapy; 4) achieving SVR, defined as an undetectable HCV load at 12 weeks after completion of anti-HCV therapy; and 5) frozen PBMC samples available to perform RNA-seq at baseline and at the end of follow-up (36 weeks after SVR). Samples and clinical data are from baseline (HIV/HCV-b) and 36 weeks after SVR (HIV/HCV-f).

Additionally, we included two control groups to compare with HIV/HCV-f group: 1) 9 Chronic HCV-monoinfected patients (HCV-mono-f) with advanced HCV-related cirrhosis who achieved SVR and had the same follow-up (36 weeks after SVR). The HCV-monoinfected patients were selected as the control group for HIV/HCV-f because HCV-monoinfected patients become patients without chronic viral infection (both HIV and HCV negative) after reaching SVR but maintaining severe liver damage. 2) 26 HIV-monoinfected patients (HIV-mono) with undetectable HIV viral load and CD4^+^ >500 cells/µL (normal standard for HIV-infected patients). We selected the HIV-monoinfected patients as the control group for HIV/HCV-f because HIV/HCV-coinfected patients after reaching SVR become HIV-monoinfected patients.

### Clinical Data And Samples

We collected clinical data prospectively using an online form. Later, the information was monitored to verify that the data collected matched the patient record. We calculated the CTP score from five factors (total bilirubin, international normalized ratio, albumin, ascites, and encephalopathy), ranging from 5 to 15 points. Trained operators assessed LSM using transient elastography (FibroScan^®^, Echosens, Paris, France), as previously described (46). LSM ranged from 2.5 to 75 kPa. After overnight fasting, the hemodynamic study to measure the HVPG was performed under light sedation with intravenous midazolam, as we previously described (47). The HVPG (expressed in mmHg) was the difference between wedged hepatic venous pressure and free hepatic venous pressure.

Peripheral venous blood samples were collected by venipuncture in ethylenediaminetetraacetic acid (EDTA) tubes. The same day, samples were sent to the HIV HGM BioBank (http://hivhgmbiobank.com/?lang=en) and were immediately processed by Ficoll-Paque density gradient, and PBMCs were stored in cryopreservation conditions (-180°C) in liquid nitrogen until analysis. Plasma was stored at -80°C.

### Multiplex ELISA Assays

Plasma biomarkers (PD1, programmed cell death ligand 1 (PDL1), C-X-C motif chemokine ligand (CXCL)10, CXCL8, C-C motif chemokine ligand (CCL)2, IFNγ, interleukin (IL)12p70, IL2, IL10, and transforming growth factor-beta (TGFβ)) were evaluated with ProcartaPlex assays (Thermo Fisher Scientific Inc, Waltham, MA, USA) using a Bio-plex 200™ system (BioRad Laboratories Hercules, CA, USA) and according to the manufacturer’s specifications. As a high proportion of samples evaluated were below the lower limit of detection for absolute quantification, we used the raw fluorescence intensity values (a.u., arbitrary units) as a relative quantification of the analyte abundances, as previously described (48).

### RNA Extraction, Library Preparation, and Sequencing

After blood extraction, PBMCs were isolated, and the RNeasy Minikit (Qiagen, Hilden, Germany) was used for total RNA purification. The RNA quantity and quality were assessed by Nanodrop 2000 and 2100 bioanalyzer RNA NANO assay (Agilent Technologies, CA, USA). The samples with an RNA integrity number over 7.5 were selected for sequencing. RNA samples were treated with RNase-free DNase set (Qiagen) following the manufacturer’s instructions. Library generation and sequencing of poly-A RNA were performed at the Centre for Genomic Regulation in Barcelona (Spain). Briefly, 500 nanograms of total RNA were used with Illumina’s TruSeq Stranded mRNA Sample Prep Kit v2 for library synthesis. This process allows capturing polyadenylated coding and non-coding RNAs. Ten libraries were multiplexed and pooled on each line of an Illumina HiSeq2500 sequencer, and a single read of 50 nts (1x50) was performed. We obtained an average of 25 million reads per sample.

Sequences were analyzed with a bioinformatic pipeline described in **Supplementary Table 1**. Briefly, for the read quality control, FastQC (v. 0.11.8) was used. Then, Trimmomatic (v. 0.38) was implemented for trimming the adapters. Reads were aligned to GRCH38 with STAR (v. 2.6.1d) software, and the Subread package of the FeatureCounts (v. 1.6.4) software was used for gene count estimation.

### Genes Selected for Analysis

**Figure 1:**
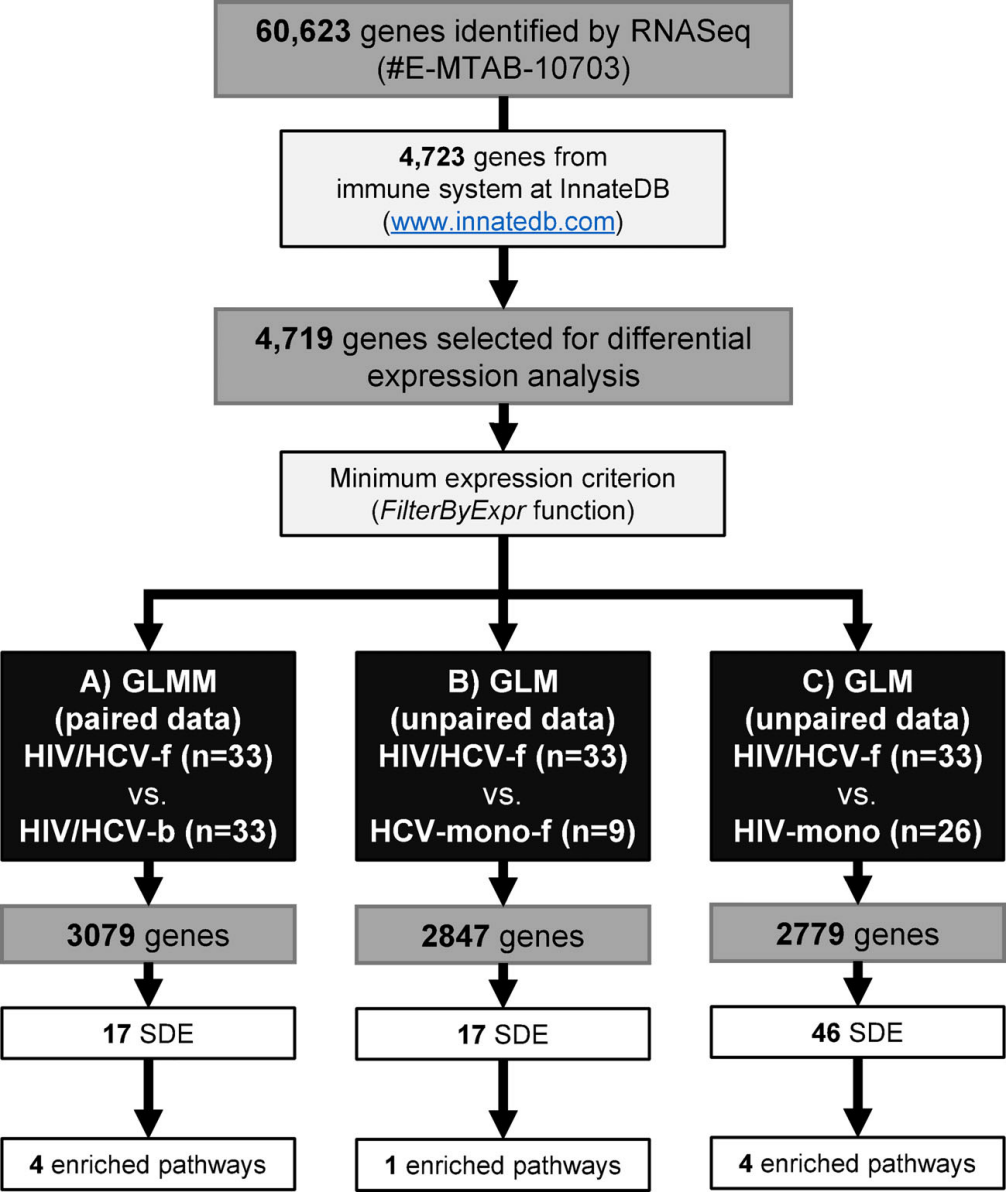
Study design flowchart. RNA sequencing identified a total of 60,623 genes. Of these, we selected 4,723 genes of the immune system from the InnateDB. A total of 4,719 genes were selected for the differential expression analysis. Next, the FilterByExpr function was applied for each comparison: **(A)** HIV/HCV-f *vs.* HIV/HCV-b, 3,079 genes were selected, 17 were SDE genes involved in four biological pathways; **(B)** HIV/HCV-f *vs.* HCV-mono-f, 2,847 genes were selected, 17 were SDE genes involved in one biological pathway; **(C)** HIV/HCV-f *vs.* HIV-mono, 2,779 genes were selected, 46 were SDE genes involved in four biological pathways. HIV, human immunodeficiency virus; HCV, hepatitis C virus; HIV/HCV-f, HIV/HCV-coinfected patients 36 weeks after the sustained virological response (SVR); HCV-mono-f, HCV-monoinfected patients 36 weeks after SVR; HIV-mono, HIV-monoinfected patients; SDE, significantly differentially expressed; GLM, generalized linear model; GLMM, Generalized linear mixed model.

**Figure 1** illustrates the workflow for the gene selection process. The bioinformatics pipeline identified a total of 60,623 different genes. Sequences are publicly available at ArrayExpress repository (EMBL-EBI; https://www.ebi.ac.uk/arrayexpress/) in raw format with the accession number E-MTAB-10703. Next, we selected 4,723 genes related to the immune system according to the public InnateDB database (www.innatedb.com), and 4,719 (99.9%) genes were identified in our sample database (**Figure 1**).

### Statistical Analysis

The Statistical Package for the Social Sciences (SPSS) v22.0 software (IBM Corp., Chicago, IL, USA) was used to describe epidemiological and clinical variables among study groups at baseline and the end of follow-up.

We carried out the gene expression analysis using the R statistical package version v. 4.0.2 (R Foundation for Statistical Computing, Vienna, Austria). First, we normalized the counts by the Trimmed Mean of M (TMM) method for each of the comparisons and established an expression filter using the *FilterByExpr* function (R-package edgeR v. 3.32.1). Next, we analyzed the gene expression between groups by generalized linear models (GLM) for non-paired comparison and generalized linear mixed models (GLMM) for paired samples, using the R-package *lme4* V. 1.1-23 and a negative binomial distribution. The differences between groups were evaluated by the fold-change (FC) and log_2_-FC for each gene. All *p-values* were two-tailed and corrected using the false discovery rate (FDR) with the Benjamini and Hochberg method (*q*-values) for multiple testing. We selected the significantly differentially expressed (SDE) genes according to FDR ≤0.05 and log_2_-FC ≤ -1 (2-fold downregulated) or log_2_-FC ≥1 (2-fold upregulated). Finally, we used TargetMine v2102 (https://targetmine.mizuguchilab.org/) to find enriched pathways and networks.

GLMM test was also used to evaluate the association between changes in biomarkers (plasma and PBMC gene expression) and liver disease scores during the follow-up in HIV/HCV-coinfected patients. The regression coefficient (β) gives the effect’s size and direction according to a positive or negative value.

## Results

### Patient Characteristics

**Table 1** shows the epidemiological and clinical characteristics at baseline of 33 HIV/HCV-coinfected patients with advanced HCV-related cirrhosis, nine HCV-monoinfected patients, and 26 HIV-monoinfected patients. All patients infected with HIV were on ART and had an undetectable HIV viral load (<50 copies/mL).

**Table 1:** Summary of patient characteristics at baseline.

	HIV/HCV-coinfected	HCV-mono	HIV-mono
**No.**	33	9	26
**Gender (male)**	25 (75.8%)	7 (77.8%)	16 (61.5%)
**Age (years)**	51.5 (48.8; 53.1)	63.5 (55.8; 71.2)	51 (46; 53)
**High alcohol intake**			
** Previously (≥ 6 months)**	15 (45.5%)	6 (66.7%)	0 (0%)
** Currently**	14 (42.4%)	3 (33.3%)	1 (3.8%)
**IVDU**			
** Previously (≥ 6 months)**	27 (81.8%)	0 (0%)	0 (0%)
** Currently**	0 (0%)	0 (0%)	0 (0%)
**Treatments**			
** Previous IFNα therapy**	15 (45.5%)	8 (88.9%)	–
** DAAs regimens**			
** Sofosbuvir + Ledipasvir**	14 (42.4%)	3 (33%)	–
** Sofosbuvir + Daclatasvir**	9 (27.3%)	1 (11.1%)	–
** Sofosbuvir + Daclatasvir + Simeprevir**	2 (6.1%)	0 (0%)	–
** Sofosbuvir + Simeprevir**	7 (21.2%)	2 (22.2%)	–
** Ombitasvir + Paritaprevir + Ritonavir + Dasabuvir**	1 (3%)	3 (33%)	–
** Antiretroviral therapy**			
** NRTI+NNRTI-based**	5 (15.2%)	–	16 (61.5%)
** NRTI+II-based**	13 (39.4%)	–	2 (7.7%)
** NRTI+PI-based**	3 (9.1%)	–	0 (0%)
** PI+II+others-based**	4 (12.1%)	–	0 (0%)
** Others**	8 (24.2%)	–	8 (30.8%)
**HIV markers**			
** Prior AIDS**	13 (39.4%)	–	10 (38.5%)
** Nadir CD4^+^ T-cells (cells/mm^3^)**	104.7 (70; 150)	–	204 (99; 343)
** Nadir CD4^+^ T-cells <200 cells/mm^3^**	27 (90%)	–	11 (44%)
** CD4^+^ T-cells (cells/mm^3^)**	393 (234; 764)	–	818 (685; 1036)
** CD4^+^ T-cells <500 cells/mm^3^**	19 (57.6%)	–	0 (0%)
** HIV-RNA >50 copies/mL**	0 (0%)	–	0 (0%)
**HCV markers**			
** HCV genotype**			
** 1**	22 (68.8%)	8 (88.9%)	–
** 3**	3 (9.4%)	1 (11.1%)	–
** 4**	7 (21.9%)	0 (0%)	–
** Log_10_ HCV-RNA (IU/mL)**	6.3 (5.8; 6.7)	6 (5.5; 6.2)	**-**
** HCV-RNA ≥850.000 IU/mL**	24 (72.7%)	5 (55.6%)	–
**Liver disease**			
** LSM (kPa)**	26.7 (19.2; 39.5)	32.6 (22.3; 64)	–
** HVPG (mmHg)**	14,5 (10; 17)	17 (10; 20)	–

Values are expressed as absolute number (percentage) and median (interquartile range).

HCV, hepatitis C virus; HIV, human immunodeficiency virus; IVDU, intravenous drug user; IFNα, interferon-alpha; DAAs, direct-acting antivirals; NRTI, nucleoside analogue HIV reverse transcriptase inhibitor; NNRTI, non-nucleoside analogue HIV reverse transcriptase inhibitor; PI, protease inhibitor; II, integrase inhibitor; AIDS, acquired immune deficiency syndrome; HCV-RNA, HCV plasma viral load; LSM, liver stiffness measures; kPa, kilopascals; HVPG, hepatic venous pressure gradient; mmHg, millimeter of mercury.

### Changes in Disease Markers in HIV/HCV-Coinfected Patients After SVR

At the end of follow-up after all-oral DAA therapy, HIV/HCV-coinfected patients exhibited a significant decrease (*q*-value <0.05) in liver disease severity markers (LSM, HVPG, bilirubin, aspartate transaminase (AST), alanine aminotransferase (ALT), gamma-glutamyl transpeptidase (GGT), and alkaline phosphatase) and immune-related markers (PD1, PDL1, CXCL10, CXCL8, IL12p70, IL10, and TGFβ) (**Table 2**). By contrast, a substantial increase in hematologic biomarkers (leukocytes, neutrophils, hematocrit, hemoglobin, and platelets) was observed (**Table 2**). However, they did not show changes in CD4^+^ T-cell and CD8^+^ T-cell values and had an undetectable HIV viral load during the whole follow-up.

**Table 2:** Summary of changes in disease markers in HIV-infected patients with advanced HCV-related cirrhosis during follow-up.

	HIV/HCV-b	HIV/HCV-f	*p*-values	*q*-values
**Liver profile**				
LSM (kPa)	26.7 (18.6; 39.6)	21.1 (14.1; 31.6)	**0.010**	**0.021**
HVPG (mmHg)	14.5 (10; 17)	12 (9.5; 14)	**0.030**	**0.047**
Bilirubin (mg/dL)	1.1 (0.8; 1.4)	0.7 (0.4; 1.1)	**<0.001**	**0.001**
AST (IU/L)	70 (37.3; 101.3)	30 (25.8; 43.5)	**0.006**	**0.016**
ALT (IU/L)	54.5 (30.5; 69.5)	23 (17.5; 32.5)	**<0.001**	**<0.001**
GGT (IU/L)	111.5 (73.5; 153)	45 (30; 62)	**0.002**	**0.007**
Alkaline phosphatase (IU/L)	113 (87.3; 152.5)	84.5 (57.5; 106)	**<0.001**	**<0.001**
**Hematologic profile**				
Leukocytes (10^6^/mL)	4.2 (3.4; 5.1)	5.1 (3.5; 6.4)	**0.033**	**0.049**
Neutrophils (10^6^/mL)	2.2 (1.5; 2.7)	2.6 (2; 3.6)	**0.007**	**0.016**
Hematocrit (%)	41.6 (38.1; 46.8)	43.1 (40.4; 46.6)	0.201	0.264
Hemoglobin (g/dL)	14 (12.8; 15.7)	14.4 (13.2; 15.5)	0.308	0.335
Platelets (10^9^/L)	80 (52.5; 104.5)	91 (67.5; 118)	**0.012**	**0.023**
**Immunologic profile**				
CD4^+^ T cells (cells/mm3)	393 (233.5; 806)	347 (218; 698)	0.235	0.294
CD8^+^ T cells (cells/mm3)	610 (376.5; 916)	609 (357; 928.5)	0.940	0.979
HIV viral load >50 copies/mL (%)	0/33 (0%)	0/33 (0%)	0.999	0.999
PD1 (a.u.)	118.5 (80.8; 147.5)	61 (46.5; 81.5)	**<0.001**	**0.002**
PDL1 (a.u.)	29 (21; 43.3)	22 (17.5; 38.8)	**<0.001**	**0.002**
CXCL10 (a.u.)	1193.5 (771; 1604)	741.5 (307.8; 994)	**<0.001**	**0.002**
CXCL8 (a.u.)	127 (79.5; 188.8)	73.5 (47; 99.3)	**0.003**	**0.008**
CCL2 (a.u.)	470 (272.8; 677)	455 (267.5; 644.8)	0.292	0.332
IFNγ (a.u.)	15 (12; 26)	16 (13; 23)	0.285	0.332
IL12p70 (a.u.)	12 (10.3; 14.3)	11 (9; 13)	**0.024**	**0.040**
IL2 (a.u.)	11 (9.3; 12.5)	10 (8.8; 12.5)	0.053	0.074
IL10 (a.u.)	37 (28.8; 46.8)	29 (25.3; 34.5)	**0.020**	**0.036**
TGFβ (a.u.)	12.2 (8.9; 18.7)	8.6 (6.7; 14.2)	**0.001**	**0.004**

Values are expressed as median (P25th; P75th). Differences were calculated by Wilcoxon signed-rank test for paired samples. P-values, raw p-values; q-values, p-values corrected for multiple testing using the false discovery rate (FDR) with Benjamini and Hochberg procedure. The statistically significant differences are shown in bold.

HCV, hepatitis C virus; HIV, human immunodeficiency virus; HIV/HCV-b, HIV/HCV-coinfected at baseline; HIV/HCV-f, HIV/HCV-coinfected at week 36 after SVR; LSM, liver stiffness measure; kPa, kilopascal; HVPG, hepatic venous pressure gradient; mmHg, millimeter of mercury; AST, aspartate transaminase; ALT, alanine aminotransferase; GGT, gamma-glutamyl transpeptidase; g, gram; mg, milligram; L, liter, dL, deciliter; mL, milliliter; IU, international unit; a.u., arbitrary units of fluorescence; PDL1, programmed death-ligand 1; PD1, programmed death-1; CXCL10, C-X-C Motif Chemokine Ligand 10; CXCL8, C-X-C Motif Chemokine Ligand 8; CCL2, C-C Motif Chemokine Ligand 2; IFNγ, interferon-gamma; IL, interleukin; TGFβ, transforming growth factor-beta.

**Figure 2:**
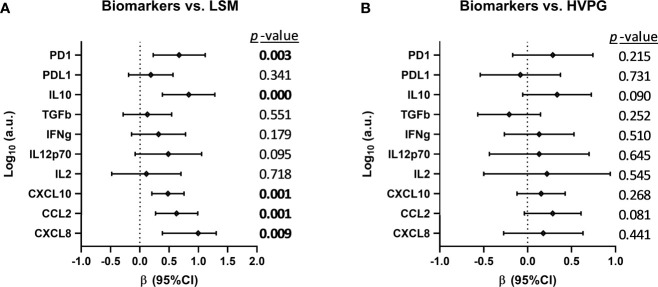
Association between changes in plasma biomarkers and liver disease severity scores **(A)** [liver stiffness measurement (LSM) and **(B)** hepatic venous pressure gradient (HVPG)] after successful all-oral direct-acting antiviral treatment in HIV/HCV-coinfected patients with advanced cirrhosis. Data were calculated by GLMM models. Values are expressed as regression coefficient (β) and 95% of confidence interval (95%CI). The statistically significant differences are shown in bold.

We analyzed the association between changes in plasma biomarkers and liver disease severity scores (LSM and HVPG) during the follow-up (**Figure 2**), but we only found a positive association (*p*-value <0.05) between plasma variations of PD1, IL10, CXCL10, CCL2, and CXCL8 and the change in LSM values (**Figure 2A**). We also performed this association analysis in HCV-monoinfected patients (see **Supplementary Figure 1**). In this case, we found a positive association (*p*-value <0.05) between plasma variations of IL-2, CXCL10, and CXCL8 and the change in LSM values, and between CXCL8 and HVGP values.

### Changes in Gene Expression in HIV/HCV-Coinfected Patients After SVR

**Figure 3:**
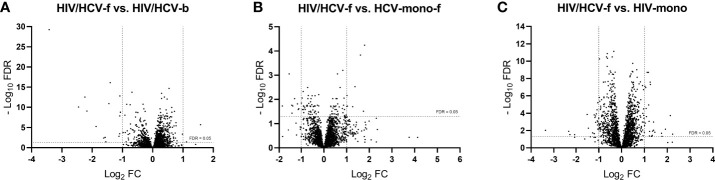
Volcano plots showing differentially expressed genes between groups: **(A)** HIV/HCV-f *vs.* HIV/HCV-b, **(B)** HIV/HCV-f *vs.* HCV-mono-f, and **(C)** HIV/HCV-f *vs.* HIV-mono. The vertical lines represent the cut-off of FC=2, and the horizontal line indicates the cut-off of FDR=0.05. FDR, false discovery rate for multiple comparisons using Benjamini and Hochberg procedure; FC, fold-change; HIV, human immunodeficiency virus; HCV, hepatitis C virus; HIV/HCV-f, HIV/HCV-coinfected patients 36 weeks after the sustained virological response (SVR); HCV-mono-f, HCV-monoinfected patients 36 weeks after SVR; HIV-mono, HIV-monoinfected patients.

A total of 3,079 genes fulfilled the expression criteria for the statistical analysis, but only 17 SDE genes were found following all-oral DAA therapy (**Figure 3A**; full description in **Table 3A**). The HIV/HCV-f group showed two upregulated (*HAS1* and *IRG1*) and 15 downregulated (*CXCL11, CCL8, CCL7, CCL2, ADARB2, RRAD, MX1, SIGLEC1, IFI44L, IFI44, IFI27, IFI6, IFIT3, IFIT1B*, and *IFIT1*) genes compared to the HIV/HCV-b group. Most of these downregulated genes were chemokines or ISGs that were classified into four significantly enriched Kyoto encyclopedia of genes and genomes (KEGG) pathways (*q*-value <0.05) (**Table 4A**): “cytokine-cytokine receptor interaction”, “viral protein interaction with cytokine and cytokine receptor”, “chemokine signaling pathway”, and “hepatitis C”.

**Table 3:** Summary of significant differentially expressed genes (absolute fold-change ≥2; FDR ≤0.05) in peripheral blood mononuclear cells between: A) HIV/HCV-coinfected at week 36 after SVR (HIV/HCV-f) versus HIV/HCV-coinfected at baseline (HIV/HCV-b). B) HIV/HCV-coinfected at week 36 after SVR (HIV/HCV-f) versus HCV-monoinfected at week 36 after SVR (HCV-mono-f); C) HIV/HCV-coinfected at week 36 after SVR (HIV/HCV-f) and HIV-monoinfected (HIV-mono).

A) HIV/HCV-f versus HIV/HCV-b					B) HIV/HCV-f versus HCV-mono-f					C) HIV/HCV-f versus HIV-mono				
Gene symbol	FC	Log_2_ (FC)	*p*-value *	*q*-value **	Gene symbol	FC	Log_2_ (FC)	*p*-value *	*q*-value **	Gene symbol	FC	Log_2_ (FC)	*p*-value *	*q*-value **
***HAS1***	2.99	1.58	≤ 0.001	≤ 0.001	***CD8A***	3.46	1.79	≤ 0.001	≤ 0.001	***IL1A***	4.72	2.24	0.009	0.026
***IRG1***	2.16	1.11	0.009	0.036	***TRGV2***	3.35	1.75	≤ 0.001	0.031	***TNFAIP6***	4.39	2.13	≤0.001	≤0.001
***IFIT3***	0.48	-1.07	≤0.001	≤0.001	***CD8B***	3.06	1.61	≤0.001	≤0.001	***SERPINB2***	3.79	1.92	0.002	0.007
***MX1***	0.47	-1.08	≤0.001	≤0.001	***LAG3***	2.59	1.37	≤0.001	0.003	***OLR1***	3.41	1.77	0.019	0.048
***IFI6***	0.47	-1.09	≤0.001	≤0.001	***TNFRSF13C***	2.41	1.27	≤0.001	0.020	***CCL20***	3.36	1.75	0.007	0.021
***ADARB2***	0.47	-1.09	≤0.001	≤0.001	***KLRK1***	2.05	1.03	≤0.001	0.019	***SIK1***	2.75	1.46	0.005	0.016
***RRAD***	0.46	-1.11	≤0.001	0.001	***IFNLR1***	2.02	1.02	≤0.001	0.007	***CXCL8***	2.65	1.41	0.002	0.009
***IFI44***	0.38	-1.41	≤0.001	≤0.001	***CDKN2A***	2.01	1.01	≤0.001	0.019	***KCNMA1***	2.64	1.40	≤0.001	0.001
***IFIT1***	0.37	-1.45	≤0.001	≤0.001	***CD180***	0.47	-1.10	≤0.001	0.024	***TNFSF15***	2.57	1.36	0.003	0.012
***IFIT1B***	0.34	-1.55	0.009	0.035	***CDKN1C***	0.46	-1.13	≤0.001	0.026	***CXCL3***	2.54	1.34	0.013	0.036
***CCL7***	0.34	-1.57	≤0.001	0.003	***FCGR3A***	0.45	-1.17	≤0.001	0.015	***LAMB3***	2.50	1.32	0.019	0.047
***CCL8***	0.33	-1.62	0.001	0.004	***SEMA3A***	0.41	-1.29	≤0.001	0.009	***CFD***	2.40	1.27	≤0.001	≤0.001
***CCL2***	0.27	-1.87	≤0.001	≤0.001	***NRXN2***	0.39	-1.37	≤0.001	0.019	***CD68***	2.39	1.26	≤0.001	≤0.001
***SIGLEC1***	0.22	-2.17	≤0.001	≤0.001	***CEACAM3***	0.38	-1.41	0.001	0.037	***EDN3***	2.34	1.23	0.004	0.013
***IFI44L***	0.21	-2.24	≤0.001	≤0.001	***CX3CR1***	0.37	-1.42	≤0.001	0.017	***DSP***	2.33	1.22	0.016	0.042
***CXCL11***	0.18	-2.45	≤0.001	≤0.001	***FAT4***	0.35	-1.53	≤0.001	≤0.001	***LRG1***	2.31	1.21	≤0.001	≤0.001
***IFI27***	0.09	-3.42	≤0.001	≤0.001	***PPP1R17***	0.28	-1.86	≤0.001	0.019	***FOXD2***	2.28	1.19	≤0.001	≤0.001
										***CCDC85B***	2.26	1.18	≤0.001	≤0.001
										***FANCL***	2.18	1.12	≤0.001	≤0.001
										***RNF144B***	2.13	1.09	≤0.001	≤0.001
										***FKBP1C***	2.11	1.08	≤0.001	≤0.001
										***CDKN2A***	2.08	1.06	≤0.001	≤0.001
										***CAVIN3***	2.06	1.04	≤0.001	0.001
										***ADAT3***	2.03	1.02	≤0.001	≤0.001
										***TNFSF9***	2.01	1.01	0.001	0.003
										***TRAV25***	0.50	-1.00	≤0.001	≤0.001
										***EDAR***	0.49	-1.04	0.002	0.007
										***PARD3***	0.49	-1.04	≤0.001	0.001
										***CNTNAP1***	0.49	-1.04	≤0.001	≤0.001
										***PF4V1***	0.48	-1.05	≤0.001	0.002
										***CMTM5***	0.48	-1.06	≤0.001	0.001
										***HAVCR1***	0.48	-1.07	≤0.001	≤0.001
										***ALOX12***	0.47	-1.10	≤0.001	≤0.001
										***FSTL1***	0.46	-1.13	≤0.001	≤0.001
										***PDZD2***	0.45	-1.14	≤0.001	≤0.001
										***PF4***	0.45	-1.17	≤0.001	≤0.001
										***IGKV1-5***	0.44	-1.19	0.007	0.023
										***GP1BA***	0.44	-1.20	≤0.001	≤0.001
										***CLEC4F***	0.43	-1.20	0.007	0.022
										***COL5A3***	0.41	-1.27	0.006	0.019
										***ADARB2***	0.36	-1.46	0.001	0.005
										***ROBO1***	0.35	-1.51	≤0.001	≤0.001
										***IGLV8-61***	0.24	-2.06	0.010	0.030
										***IGLV2-23***	0.20	-2.29	0.010	0.029
										***IGLV2-18***	0.20	-2.34	0.004	0.012
										***IGHG2***	0.10	-3.37	0.003	0.010

Values are expressed as fold-change (FC) and its log_2_. (*), raw p-values; (**), p-values corrected for multiple testing using the false discovery rate (FDR) with Benjamini and Hochberg procedure.

HCV, hepatitis C virus; HIV, human immunodeficiency virus; HIV/HCV-f, HIV/HCV-coinfected at week 36 after SVR; HIV/HCV-b, HIV/HCV-coinfected at baseline; HCV-mono-f, HCV-monoinfected at week 36 after SVR; HIV-mono, HIV-monoinfected patients.

**Table 4:** Summary of significant pathways (FDR ≤0.05) in peripheral blood mononuclear cells between: A) HIV/HCV-coinfected at week 36 after SVR (HIV/HCV-f) versus HIV/HCV-coinfected at baseline (HIV/HCV-b). B) HIV/HCV-coinfected at week 36 after SVR (HIV/HCV-f) versus HCV-monoinfected at week 36 after SVR (HCV-mono-f); C) HIV/HCV-coinfected at week 36 after SVR (HIV/HCV-f) and HIV-monoinfected (HIV-mono).

A) HIV/HCV-f versus HIV/HCV-b				B) HIV/HCV-f versus HCV-mono-f				C) HIV/HCV-f versus HIV-mono			
KEGG	Hits	Genes	*q*-values	KEGG	Hits	Genes	*q*-values	KEGG	Hits	Genes	*q*-values
Cytokine-cytokine receptor interaction	4	CCL2, CCL7, CCL8, CXCL11	0.021	Primary immunodeficiency	3	CD8A, CD8B, TNFRSF13C	0.009	Cytokine-cytokine receptor interaction	9	CCL20, CXCL3, CXCL8, EDAR, IL1A, PF4, PF4V1, TNFSF15, TNFSF9	<0.001
Viral protein interaction with cytokine and cytokine receptor	4	CCL2, CCL7, CCL8, CXCL11	0.001					Viral protein interaction with cytokine and cytokine receptor	5	CCL20, CXCL3, CXCL8, PF4, PF4V1	0.002
Chemokine signaling pathway	4	CCL2, CCL7, CCL8, CXCL11	0.006					Chemokine signaling pathway	6	CCL20, CXCL3, CXCL8, PARD3, PF4, PF4V1	0.003
Hepatitis C	3	IFIT1, IFIT1B, MX1	0.047					Rheumatoid arthritis	4	CCL20, CXCL3, CXCL8, IL1A	0.017

q-values, p values corrected for multiple comparisons using Benjamini and Hochberg procedure. In red, upregulated genes in HIV/HCV-f group; in green, downregulated genes in HIV/HCV-f group.

KEGG, Kyoto Encyclopedia of Genes and Genomes; HIV, human immunodeficiency virus; HCV, hepatitis C virus; HIV/HCV-b, HIV/HCV-coinfected patients at baseline; HIV/HCV-f, HIV/HCV-coinfected patients 36 weeks after SVR; HCV-mono-f, HCV-monoinfected at week 36 after SVR; HIV-mono, HIV-monoinfected patients.

**Figure 4:**
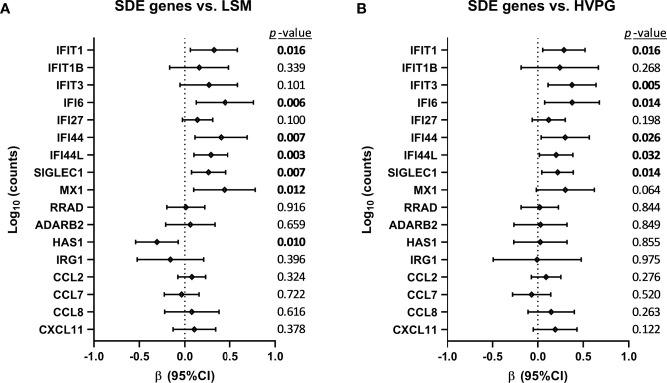
Association between changes in significant differentially expressed (SDE) genes and liver disease severity scores **(A)** [liver stiffness measurement (LSM) and **(B)** hepatic venous pressure gradient (HVPG)] after successful all-oral direct-acting antiviral treatment in HIV/HCV-coinfected patients with advanced cirrhosis. Data were calculated by GLMM models. Values are expressed as regression coefficient (β) and 95% of confidence interval (95%CI). The statistically significant differences are shown in bold.

The association between changes in SDE genes and liver disease severity scores (LSM and HVPG) is shown in **Figure 4**. We found positive associations (*p*-value <0.05) of *IFIT1*, *IFI6*, *IFI44*, *IFI44L*, *SIGLEC1*, and *MX1* with LSM values, and *HAS1* showed a negative association (**Figure 4A**). Additionally, we found positive associations (*p*-value <0.05) of *IFIT1, IFIT3, IFI6, IFI44, IFI44L*, and *SIGLEC1* with HVPG values (**Figure 4B**).

We also evaluated SDE genes in HCV-monoinfected patients (see **Supplementary Table 2**). In this case, we found 4 SDE (*HAS1, IFI44L, SIGLEC1*, and *IFI27*), which were also differentially expressed in HIV/HCV-coinfected patients after SVR. However, we also found seven other SDE genes (*SNAI1*, *MAPK8IP1*, *SHB*, *IFNG*, *NRCAM*, *ZBTB32*, and *CYP1A1*) that are ISGs.

### Gene Expression in HIV/HCV-Coinfected Patients After SVR Compared to Control Groups

By comparing the HIV/HCV-f with the HCV-mono-f, 2,847 genes met the expression criteria, and 17 SDE genes were found (**Figure 3B**, full description in **Table 3B**). The HIV/HCV-f group had eight upregulated and nine downregulated genes compared to the HCV-mono-f group. The analysis of the altered pathways related to these genes (**Table 4B**) showed the only significant KEGG pathway identified was “primary immunodeficiency pathway” (*q*-value <0.05), with three SDE genes upregulated (*CD8A, CD8B*, and *TNFRSF13C*) in HIV/HCV-f.

In the HIV/HCV-f versus HIV-mono comparison, 2,779 genes met the expression criteria, and 46 SDE genes were found (**Figure 3C**, full description in **Table 3C**). The HIV/HCV-f group had 25 upregulated and 21 downregulated genes compared with the HIV-mono group. The analysis of the altered pathways (**Table 4C**) gave four significant results (*q*-value <0.05) in the KEGG enrichment analysis (“cytokine-cytokine receptor interaction”, “viral protein interaction with cytokine and cytokine receptor”, “chemokine signaling pathway”, and “rheumatoid arthritis”), with six SDE genes upregulated (*CCL20, CXCL3, CXCL8, IL1A, TNFSF15*, and *TNFSF9*) and four downregulated (*EDAR, PF4, PARD3*, and *PF4V1*) in HIV/HCV-f. The HIV/HCV-f *vs.* HCV-mono-f and the HIV/HCV-f *vs.* HIV-mono comparisons showed only one upregulated gene in common (*CDKN2A*).

## Discussion

In this study, our significant findings for HIV/HCV-coinfected patients after SVR following all-oral DAA therapy were: i) an improvement in immunological and liver disease markers, with an association between decreased plasma levels of PD1, IL10, CXCL10, CXCL8, and CCL2 and a reduction in LSM values; ii) a decrease in the expression of ISGs, mainly encoding for chemokines and antiviral proteins, which were grouped into four altered pathways (“cytokine-cytokine receptor interaction”, “viral protein interaction with cytokine and cytokine receptor”, “chemokine signaling pathway”, and “hepatitis C”); additionally, the decrease in most of these genes was also related to a reduction in LSM or HVPG values; and iii) only the “primary immunodeficiency pathway” was altered compared to HCV-mono-f, while four pathways (“cytokine-cytokine receptor interaction”, “viral protein interaction with cytokine and cytokine receptor”, “chemokine signaling pathway”, and “rheumatoid arthritis”) were altered compared to HIV-monoinfected patients.

Little is known about how HCV elimination after all-oral DAA therapy affects the immune system of HIV/HCV-coinfected patients. This population is of particular interest since they suffer from higher inflammation and accelerated fibrogenesis than HCV-monoinfected patients (49, 50), and despite ART, residual inflammation remains in HIV-monoinfected patients (51, 52). Chronic hepatitis C promotes hepatic and systemic inflammation, with high production of CXCL8, CCL2, CXCL10, IFNγ, and IL12p70, among others (13), and activation and exhaustion of the immune system, with increased levels of PD1 and PDL1 (53). Our data show an improvement in these immune and liver disease biomarkers in plasma (immune exhaustion (PD1), chemokines (CXCL10, CCL2, and CXCL8), and cytokines (IL10)) after achieving SVR with DAA therapy in HIV/HCV-coinfected individuals. These results are similar to those found in HCV-monoinfected persons, in whom immune and liver function improved after HCV clearance by DAA, although normalization of the immune system after SVR is not always achieved (54, 55). Regarding HIV/HCV-coinfected patients, there are limited data on their status following DAA therapy. However, some of our findings are similar to those described in previous studies showing improved liver function (56–61) and a decrease in plasma chemokine levels (CXCL10, CCL2, and CXCL8) (45, 62–66). Chemokines attract leucocytes to the liver, promoting cirrhosis and the appearance of LRE (67), and they are surrogate markers of liver inflammation (68). Thus, the decline of CXCL10, CCL2, and CXCL8 would indicate a reduction of liver and systemic inflammation after HCV clearance. Chronic hepatitis C upregulates exhaustion markers, such as PD1 (53, 66), which is an immune checkpoint receptor that inhibits the adaptive immune response. Thus, a decrease in plasma levels of PD1 would indicate recovery of the exhausted adaptive immune response, as seen in HCV-monoinfected patients (53, 66). IL10 has a central role in chronic hepatitis C, limiting the inflammatory response (69) and reducing liver fibrosis (70). However, we found a decrease in plasma IL10 after SVR, which may be a consequence of HCV clearance and a general reduction in inflammation.

Gene expression in PBMCs was also analyzed in HIV/HCV-coinfected patients, where we mainly found a decrease in ISGs after achieving SVR with all-oral DAA therapy. ISGs are related to the degree of host immune dysregulation in chronic hepatitis C, affecting the control of virus replication and spontaneous HCV clearance (13). Chronic hepatitis C is also associated with increased levels of several relevant IFN-sensitive chemokines that promote the development of liver fibrosis and cirrhosis (67). In HCV-monoinfected patients, several studies have reported the downregulation of many chemokines, cytokines, and other ISG genes in PBMCs after SVR following DAA therapy (43, 71–75), but normal levels of these genes are not always restored, indicating incomplete normalization of the immune system. Information on the impact of HCV clearance after DAA therapy on PBMC gene expression in HIV/HCV-coinfected patients is scarce, but changes in ISGs and pathways related to antiviral defense, innate immune response, cytokine signals, and inflammatory response/chemotaxis have been previously observed (44, 45). The prolonged and sustained down-regulation of ISGs suggests a reversal of the exhausted and inflammatory immune phenotype after hepatitis C cure with all-oral DAA therapy. Furthermore, in our study, reduced ISG expression was associated with an improvement in liver disease severity scores (LSM and HVPG) in HIV/HCV-coinfected patients who achieved SVR following all-oral DAA therapy.

We also performed a statistical analysis in HCV-monoinfected patients during follow-up (baseline *vs.* after-SVR) to evaluate whether there is a pattern similar to HIV/HCV-coinfected patients. On the one hand, we found that plasma CXCL10 and CXCL8 remained positively associated with LSM values in HCV-monoinfected and HIV/HCV-coinfected patients, but IL-2 was also associated with LSM and CXCL8 with HVPG in HCV-monoinfected patients. On the other hand, 4 SDE (*HAS1, IFI44L, SIGLEC1*, and *IFI27*) were common in HCV-monoinfected and HIV/HCV-coinfected patients, but seven other SDE genes (*SNAI1*, *MAPK8IP1*, *SHB*, *IFNG*, *NRCAM*, *ZBTB32*, and *CYP1A1*) were only found in HCV-monoinfected patients; which are all ISGs (http://www.interferome.org/). These findings show that the changes in HCV monoinfected patients after SVR were not precisely the same as in HIV/HCV-coinfected patients, although almost equivalent.

Moreover, we found that the PBMC gene expression of *CD8A*, *CD8B*, and *TNFRSF13C* was higher in HIV/HCV-coinfected patients after achieving SVR (HIV/HCV-f) than HCV-monoinfected patients after SVR (HCV-mono-f). These three genes are grouped into the “primary immunodeficiency pathway”, the only KEGG pathway with a q-value lower than 0.25. The overexpression of *CD8A* and *CD8B* may be linked to increased CD8 T-cell count, an intrinsic feature of HIV infection. HIV-infected patients have the CD4/CD8 ratio usually diminished in most patients, and it is related to higher inflammation, activation, immune deregulation, and increased risk of non-AIDS morbidity and mortality (76). A similar finding was found in HIV-infected patients with sepsis, who showed overexpression of genes involved in cytotoxic T-cell signaling (*CD8A*, *CD8B*) (77). These findings reveal an effect of HIV infection *per se*, which persists after SVR. However, this does not answer why HIV/HCV-coinfected patients have higher rates of liver-related events than HCV-monoinfected patients. Besides, *TNFRSF13C* is a regulator of the peripheral B-cell population that enhances B-cell survival, which is decreased by HCV infection, and it has been related to HCV-induced B cells clonal disorders, such as mixed cryoglobulinemia and non-Hodgkin’s lymphoma (78). Also, an increase in *TNFRSF13C* expression has been described after HCV eradication with antiviral treatment, together with complete clinical remission of HCV-induced B-cell clonal disorders (78). Overexpression of *TNFRS13C* in HIV/HCV-coinfected patients may be related to continuous stimulation of B cells by HIV chronic infection. The other two genes in the top 5 of overexpression were *TRGV2* and *LAG3*. TRGV2 is a T-cell receptor gamma chain that participates in HIV control, indicating a recovery of the immune response against the virus (79). However, *LAG3* is upregulated in T-cells of HIV-monoinfected patients and point to immune exhaustion, characterized by functional unresponsiveness of T-cells (80).

HIV/HCV-f and HCV-mono-f showed no differences in the PBMC gene expression levels of chemokines and ISGs and plasma levels of biomarkers related to immune exhaustion, chemokines, and Th1 and Th2 cytokines (*data not shown*) after HCV elimination by DAA. Overall, these data indicate that HCV treatment had a similar impact on peripheral blood markers in both groups.

When compared to HIV-monoinfected individuals, some cytokine genes (*CCL20, CXCL3, CXCL8, IL1A, TNFSF15*, and *TNFSF9*) were upregulated in HIV/HCV-coinfected patients after HCV elimination. This, again, indicates that although DAA treatment leads to a broad reduction in cytokine and ISG expression, the restoration of the immune response is not complete. Particularly worrying are the elevated levels of CCL20, which is a pro-angiogenic factor that promotes HCC hypervascularization (81) and might be behind the increased probability of developing HCC following SVR (82–84). High gene expression levels of *CXCL3*, *CXCL8*, and *IL1A* also correlate with HCV-associated liver inflammation, cirrhosis, and HCC (85–89). Overexpression of *TNFSF15* increases inflammation and both liver and intestinal fibrosis, promoting chronic immunological diseases (90, 91). Moreover, we observed lower expression of *PF4* and *PF4V1* in HIV/HCV-coinfected patients after SVR than in HIV-monoinfected patients. *PF4* and *PF4V1* genes encode two chemokines (CXCL4 and CXCL4L1, respectively) related to platelet activation, and their expression promotes inflammation and wound repair. In the liver, CXCL4 and CXCL4L1 stimulate proliferation, chemotaxis, and chemokine expression of hepatic stellate cells, promoting liver fibrosis (92, 93). The lowest values found in HIV/HCV-coinfected patients after SVR may reflect HCV elimination.

### Study Limitations

Our conclusions should be interpreted with caution. i) The limited sample size can reduce the statistical power to detect small differences between groups. In addition, this can also increase the risk of false-positive results. ii) The design of our study was prospective, and biases could have been introduced. Nevertheless, the principal analysis of the study was carried out with a GLMM model for paired data, which reduces the impact of study biases, limits the false positive rate, and increases statistical power. iii) Our work was an association study and lacked mechanistic exploration or functional validation.

### Final Remarks

HCV elimination by DAA treatment in HIV/HCV-coinfected individuals improves liver inflammation and HCV-related extrahepatic diseases in most cases (94). Nevertheless, while some aspects of the immune response are restored, others remain altered. Therefore, long-term chronic HCV infection might lead to an irreversible impairment of the immune system, perhaps by inducing epigenetic changes that persist after virus eradication (95). However, since virus-host interactions are very complex, it is difficult to know which gene expression changes are clinically relevant.

Due to an unbalanced immunity after SVR, HCV elimination poses some concerns regarding reactivation of latent coinfections, such as HBV (96) or herpesvirus (97, 98), or the development of HCC (82–84). For instance, despite reducing inflammation, an increase in angiogenesis-related gene expression, which may favor HCC development, has been observed in cured patients (81, 99). Interestingly, long-term epigenetic modifications induced by HCV are associated with an increased risk for HCC development even after HCV cure with DAA (95). However, other studies have not identified any effect of DAA on the recurrence of HCC (100).

Knowing the extent and causes of the partial recovery of the immune system in HIV patients on ART after HCV chronic infection might be relevant to understanding the susceptibility of cured patients to the development of immune-mediated diseases, cancer, and infections with different pathogens (including re-infections), as well as contributing to drug design and vaccine.

### Conclusions

HIV/HCV-coinfected patients with advanced cirrhosis who eradicated HCV infection with all-oral DAA therapy exhibited significant decreases in plasma biomarkers and gene expression related to antiviral/inflammatory response, particularly several chemokines and ISGs, along with an improvement in liver disease markers. However, complete normalization of the immune system was not achieved, as observed by comparison with HCV and HIV-monoinfected patients.

## Data Availability Statement

The datasets used and analyzed during the current study are available from the corresponding authors upon reasonable request. Patients’ raw sequence data are publicly available at the ArrayExpress repository (EMBL-EBI; https://www.ebi.ac.uk/) under the accession number E-MTAB-10703.

## Ethics Statement

The studies involving human participants were reviewed and approved by The Institutional Review Board and the Research Ethics Committee of the Instituto de Salud Carlos III (ISCIII) approved the study (CEI PI 41_2014). The patients/participants provided their written informed consent to participate in this study.

## Author Contributions

Conceptualization: SR and MA, and AF-R. Data curation: JB, JG-G, CD, VH, LI-S, and LP-L. Formal analysis: OB-K, SR, IM, and AF-R. Funding acquisition: JB, JG-G, MA, and SR. Investigation and methodology: SS, OB-K, MA, and AF-R. Project administration: JB and SR. Supervision and visualization: MA and SR. Writing – original draft preparation: IM, AF-R, MA, and SR. Writing – Review and editing: DS-C. All authors contributed to the article and approved the submitted version.

## Funding

This study was supported by grants from Instituto de Salud Carlos III (ISCII; grant numbers PI20/00474 and PI17/00657 to JB, PI20/00507 and PI17/00903 to JGG, PI18CIII/00020 to AF-R, PI18CIII/00028 to MA, and PI20CIII/00004 and PI17CIII/00003 to SR). AF-R and MA are Miguel Servet researchers supported and funded by ISCIII (grant numbers: CP14CIII/00010 to AFR and CP17CIII/00007 to MAJS). The study was also funded by the RD16/0025/0017, RD16/0025/0018 and RD16CIII/0002/0002 projects as part of the Plan Nacional R + D + I and co-funded by ISCIII- Subdirección General de Evaluación and the Fondo Europeo de Desarrollo Regional (FEDER). JB is an investigator from the Programa de Intensificación de la Actividad Investigadora en el Sistema Nacional de Salud (I3SNS), Ref. INT16/00100. DS-C is a ‘Sara Borrell’ researcher from ISCIII (grant number CD20CIII/00001) and has also been supported through Fundación SEIMC-GESIDA by a fellowship award from Fundación ONCE ‘Oportunidad al Talento, 2019/20 and 2020/21’ co-financed by Fondo Social Europeo.

## Conflict of Interest

The authors declare that the research was conducted in the absence of any commercial or financial relationships that could be construed as a potential conflict of interest.

## Publisher’s Note

All claims expressed in this article are solely those of the authors and do not necessarily represent those of their affiliated organizations, or those of the publisher, the editors and the reviewers. Any product that may be evaluated in this article, or claim that may be made by its manufacturer, is not guaranteed or endorsed by the publisher.

## Appendix

**The ESCORIAL study group.**

**Hospital General Universitario Gregorio Marañón** (Madrid, Spain): Cristina Díez, Luis Ibáñez, Leire Pérez-Latorre, Diego Rincón, Teresa Aldámiz-Echevarría, Vega Catalina, Pilar Miralles, Teresa Aldámiz-Echevarría, Francisco Tejerina, María C Gómez-Rico, Esther Alonso, José M Bellón, Rafael Bañares, and Juan Berenguer.

**Hospital Universitario La Paz/IdiPAZ** (Madrid, Spain): José Arribas, José I Bernardino, Carmen Busca, Ana Delgado, Javier García-Samaniego, Víctor Hontañón, Luz Martín-Carbonero, Rafael Micán, María L Montes-Ramírez, Victoria Moreno, Antonio Olveira, Ignacio Pérez-Valero, Eulalia valencia, and Juan González-García.

**Hospital Universitario Puerta de Hierro** (Madrid, Spain): Elba Llop and José Luis Calleja.

**Hospital Universitario Ramón y Cajal** (Madrid, Spain): Javier Martínez and Agustín Albillos.

**Fundación SEIMC/GeSIDA** (Madrid, Spain): Marta de Miguel, María Yllescas, and Herminia Esteban.
