# Supplementary material for: HCV Cure With Direct-Acting Antivirals Improves Liver and Immunological Markers in HIV/HCV-Coinfected Patients
Source: Front Immunol. 2021 Aug 23;12:723196. doi: 10.3389/fimmu.2021.723196 (PMC8419228; doi:10.3389/fimmu.2021.723196)

**Supplementary Table 1.** Bioinformatics pipeline to analyze raw sequences from RNA-seq of HIV/HCV-coinfected, HIV-monoinfected, and HCV-monoinfected patients.

| *1. Filtering step*  Settings employed to eliminate adapters and low-quality reads.  - Software:  Trimmomatic  - Version: 0.38  java -jar /opt/Trimmomatic-0.33/trimmomatic-0.33.jar SE -threads 5 -phred33 sample_id _R1.fastq.gz sample_id "_filtered_R1.fastq" ILLUMINACLIP:$TRIMMOMATIC_PATH/adapters/NexteraPE-PE.fa:2:30:10 SLIDINGWINDOW:4:20 MINLEN:50 2> sample_id.log  gzip *.fastq  fastqc --quiet --threads 5 *_filtered_*.fastq.gz  touch sample_id "_unpaired_R1.fastq.gz"  mv .command.log sample_id.command.log  mv .command.sh sample_id.command.sh  mv .command.err sample_id.command.err |
| --- |
| *2. Alignment*  Settings employed for mapping the filtered counts.  - Software:  STAR  - Version:  2.6.1  - Human genome:  GRCh38  - Code:  STAR --genomeDir star_index \  --sjdbGTFfile Homo_sapiens.GRCh38.98.gtf \  --readFilesIn sample_id_filtered_R1.fastq.gz \  --runThreadN 16 \  --twopassMode Basic \  --outWigType bedGraph \  --outSAMtype BAM SortedByCoordinate --limitBAMsortRAM 7516182768 \  --readFilesCommand zcat \  --runDirPerm All_RWX \  --outFileNamePrefix sample_id_filtered  samtools index sample_id_filteredAligned.sortedByCoord.out.bam  mv .command.log sample_id_filtered.command.log  mv .command.sh sample_id_filtered.command.sh  mv .command.err sample_id_filtered.command.err |
| *3. Count step*  Settings employed to obtain the number of counts per gene and sample  - Software:  FeatureCounts from subread package  - Version:  1.6.4  - Code:  #!/bin/bash -euo pipefail  featureCounts -a Homo_sapiens.GRCh38.98.gtf -g gene_id -o sample_id_gene.featureCounts.txt -p -s 2 sample_id_filteredAligned.sortedByCoord.out.bam  featureCounts -a Homo_sapiens.GRCh38.98.gtf -g gene_biotype -o sample_id_biotype.featureCounts.txt -p -s 2 sample_id_filteredAligned.sortedByCoord.out.bam  cut -f 1,7 sample_id_biotype.featureCounts.txt \| tail -n +3 \| cat biotypes_header.txt - >> sample_id_biotype_counts_mqc.txt  mqc_features_stat.py sample_id_biotype_counts_mqc.txt -s sample_id -f rRNA -o sample_id_biotype_counts_gs_mqc.tsv  mv .command.log sample_id.command.log  mv .command.sh sample_id.command.sh  mv .command.err sample_id.command.err |

**Supplementary Table 2.** Summary of significant differentially expressed genes (absolute fold-change ≥2; FDR ≤0.05) in peripheral blood mononuclear cells between HCV-monoinfected at week 36 after SVR (HCV-f) versus HCV-monoinfected at baseline (HCV-b).

| Gene symbol | FC | Log_2_ (FC) | *p*-value * | *q*-value ** |
| --- | --- | --- | --- | --- |
| *HAS1* | 3.59 | 1.84 | 0.008 | 0.050 |
| *SNAI1* | 2.66 | 1.41 | 0.007 | 0.044 |
| *MAPK8IP1* | 0.50 | -1.01 | ≤0.001 | 0.003 |
| *SHB* | 0.48 | -1.05 | 0.007 | 0.044 |
| *IFI44L* | 0.47 | -1.10 | 0.001 | 0.008 |
| *IFNG* | 0.47 | -1.07 | ≤ 0.001 | 0.004 |
| *NRCAM* | 0.47 | -1.10 | 0.002 | 0.020 |
| *ZBTB32* | 0.45 | -1.17 | ≤0.001 | ≤0.001 |
| *SIGLEC1* | 0.40 | -1.33 | 0.002 | 0.016 |
| *IFI27* | 0.05 | -4.35 | ≤0.001 | ≤0.001 |
| *CYP1A1* | 0.05 | -4.25 | ≤0.001 | 0.002 |

**Statistics**: Values are expressed as fold-change (FC) and its log_2_. (*), raw *p*-values; (**), *p*-values corrected for multiple testing using the false discovery rate (*FDR*) with Benjamini and Hochberg procedure.

**Abbreviations**. HCV, hepatitis C virus

**Supplementary Figure 1.** Association between changes in plasma biomarkers and liver disease severity scores (liver stiffness measurement (LSM) and hepatic venous pressure gradient (HVPG)) after successful all-oral direct-acting antiviral treatment in HCV-monoinfected patients with advanced cirrhosis.

**Statistics**: Data were calculated by GLMM models. Values are expressed as regression coefficient (β) and 95% of confidence interval (95%CI). The statistically significant differences are shown in bold.


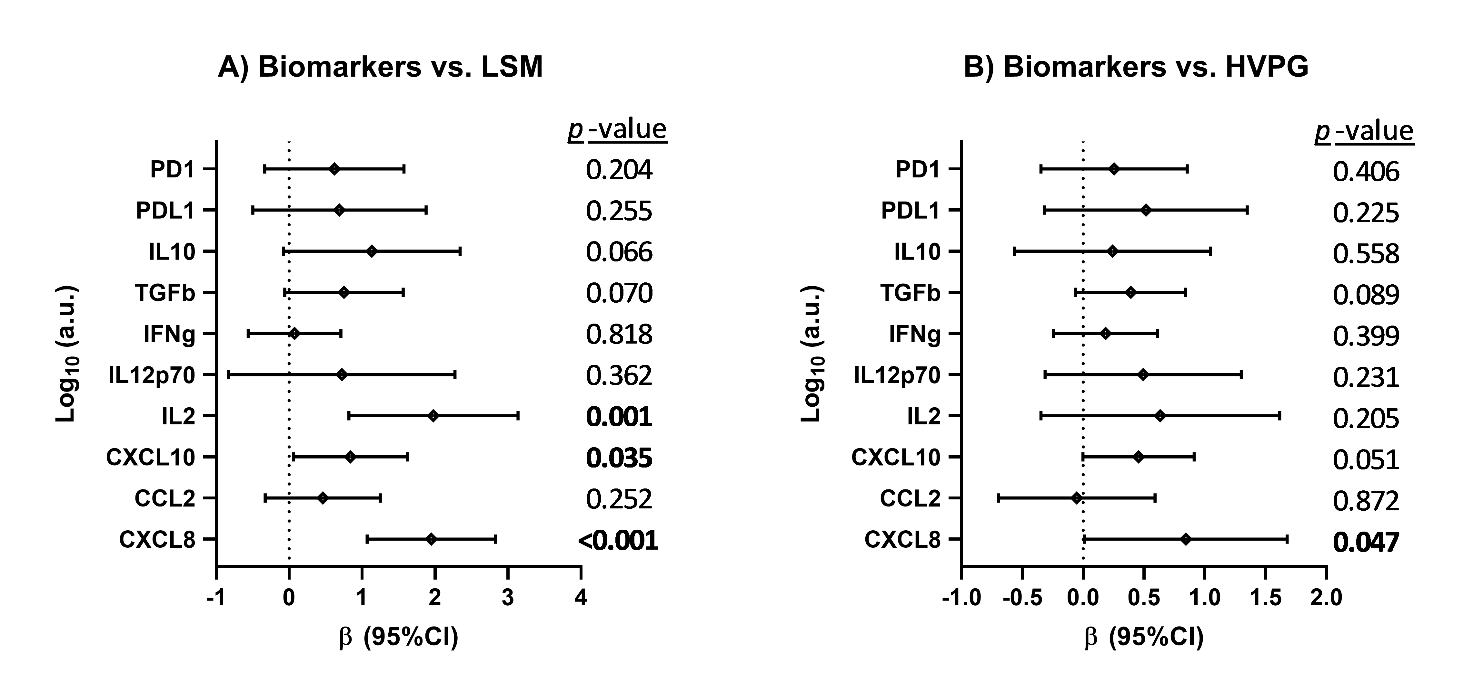

Supplement: Supplementary file 1 [file Table_1.docx]
